# Beneficial Effects of Dietary Polyphenols on Gut Microbiota and Strategies to Improve Delivery Efficiency

**DOI:** 10.3390/nu11092216

**Published:** 2019-09-13

**Authors:** Amit Kumar Singh, Célia Cabral, Ramesh Kumar, Risha Ganguly, Harvesh Kumar Rana, Ashutosh Gupta, Maria Rosaria Lauro, Claudia Carbone, Flávio Reis, Abhay K. Pandey

**Affiliations:** 1 Department of Biochemistry, University of Allahabad, Allahabad 211002, India; amitfbs21@gmail.com (A.K.S.); rameshbiochem91@gmail.com (R.K.); rishaganguly53@gmail.com (R.G.); harshversity@gmail.com (H.K.R.); ashutosh8998@gmail.com (A.G.); 2 Coimbra Institute for Clinical and Biomedical Research (iCBR), Faculty of Medicine; & CIBB Consortium, University of Coimbra, 3000-548 Coimbra, Portugal; celia.cabral@fmed.uc.pt; 3 Laboratory of Pharmaceutical Technology, Department of Pharmacy, University of Salerno, 84084 Fisciano (SA), Italy; lauro@unisa.it; 4 Laboratory of Drug Delivery Technology, Department of Drug Sciences, University of Catania, 95125 Catania, Italy; ccarbone@unict.it; 5 Institute of Pharmacology & Experimental Therapeutics, Faculty of Medicine; University of Coimbra, 3000-548 Coimbra, Portugal

**Keywords:** dietary polyphenols, gut microbiota, dysbiosis, gastrointestinal diseases, metabolic disorders, delivery systems

## Abstract

The human intestine contains an intricate ecological community of dwelling bacteria, referred as gut microbiota (GM), which plays a pivotal role in host homeostasis. Multiple factors could interfere with this delicate balance, including genetics, age, antibiotics, as well as environmental factors, particularly diet, thus causing a disruption of microbiota equilibrium (dysbiosis). Growing evidences support the involvement of GM dysbiosis in gastrointestinal (GI) and extra-intestinal cardiometabolic diseases, namely obesity and diabetes. This review firstly overviews the role of GM in health and disease, then critically reviews the evidences regarding the influence of dietary polyphenols in GM based on preclinical and clinical data, ending with strategies under development to improve efficiency of delivery. Although the precise mechanisms deserve further clarification, preclinical and clinical data suggest that dietary polyphenols present prebiotic properties and exert antimicrobial activities against pathogenic GM, having benefits in distinct disorders. Specifically, dietary polyphenols have been shown ability to modulate GM composition and function, interfering with bacterial quorum sensing, membrane permeability, as well as sensitizing bacteria to xenobiotics. In addition, can impact on gut metabolism and immunity and exert anti-inflammatory properties. In order to overcome the low bioavailability, several different approaches have been developed, aiming to improve solubility and transport of dietary polyphenols throughout the GI tract and deliver in the targeted intestinal regions. Although more research is still needed, particularly translational and clinical studies, the biotechnological progresses achieved during the last years open up good perspectives to, in a near future, be able to improve the use of dietary polyphenols modulating GM in a broad range of disorders characterized by a dysbiotic phenotype.

## 1. Introduction

Hippocrates has been cited as saying “death sits in the bowels” and “bad digestion is the root of all evil” in around 400 B.C., suggesting the important role of the human intestine in health and disease. Gut microbiota (GM) is the collective community of microorganisms living in the gastrointestinal (GI) tract. Approximately 100 trillions of microorganisms, consisting mainly of bacteria, inhabits in the human GI tract [1]. Viruses, protozoa and eukaryotic organisms, such as fungi, are also present in a small number. In the adult GI tract about 90% of the bacteria fit in the phyla Bacteroidetes (Gram-negative) and Firmicutes (Gram-positive), while other phyla are present in much lower abundance, such as Actinobacteria (Gram-positive), namely *Bifidobacterium*, Proteobacteria (Gram-negative) and Verrucomicrobia (Gram-negative), namely *Akkermansia muciniphila* (Gram-negative) [1,2].

**Figure 1:**
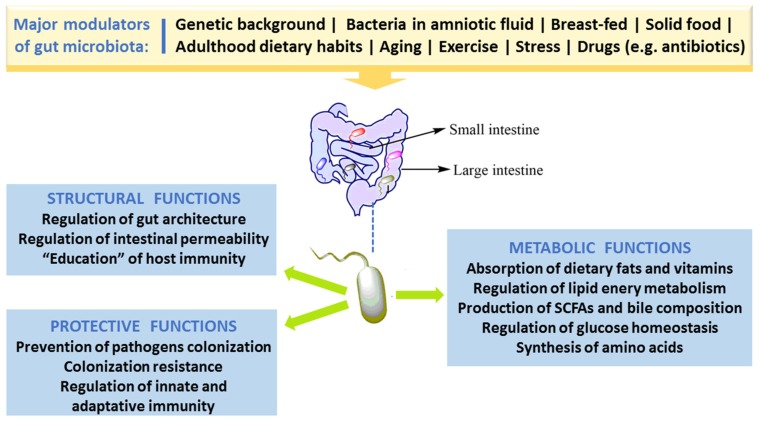
Major factors that modulate gut microbiota and key roles in humans.

In spite of the fact that people have several hundreds of microbial species inside their gut, newer findings obtained by the Human Microbiome Project and other relevant studies demonstrate that microbial composition is highly variable between individuals [3]. Bacterial colonization starts in utero and GM composition changes throughout the entire life, but the main changes, in number and in diversity, occur during the breast-feeding period and at the beginning of solid food ingestion. The number, type and function of microorganisms differ throughout the GI tract but the bulk is found inside the large intestine, participating in fermentation of undigested food components, particularly carbohydrates and fibers, among other relevant functions. The main roles of GM in humans are depicted in Figure 1. Apart from affording protection against enteropathogens and absorb nutrients from our diet, GM produces several bioactive compounds, some of which are beneficial to health, namely vitamins and some short chain fatty acids (SCFAs), while others are deleterious, such as some metabolites of degradation of amino acids. In addition, host immune defenses, in particular the mucus barrier, are important to protect tissues against harmful effects of some bacteria.

Factors responsible for an impaired GM composition and/or function, called dysbiosis, include age, diet and lack of exercise, stress, drugs and xenobiotics [1,4,5]. There is increasing evidence supporting an association between dysbiosis and diseases, including those of the GI tract, such as inflammatory bowel disease (IBD), ulcerative colitis (UC), Crohn disease (CD) and colorectal cancer (CRC) [6,7,8], as well as some extra-intestinal metabolic disorders, including obesity, diabetes and its macro- and microvascular complications [9,10,11]. Hence, researchers all over the world are searching for therapeutic or nutraceutical interventions able to produce a healthy GM equilibrium, eliminating the harmful bacteria (or pathobionts) without affecting the beneficial ones (symbionts).

Dietary polyphenols are natural compounds present in many foods and beverages, namely in fruits, vegetables, cereals, tea, coffee, and wine, among others. Several preclinical and clinical studies have shown their antioxidant, anti-inflammatory, anti-diabetic, anti-cancer, neuroprotective, and anti-adipogenic properties, suggesting a link between polyphenol-rich food consumption and reduction in the incidence of numerous chronic disorders, highlighting them as good candidates for therapeutic/nutraceutical agents [12,13,14,15]. However, inside the human body, the chemical structure of the majority of polyphenols is received as a xenobiotic and, thus, the bioavailability of these compounds is highly reduced when compared to that of macro- and micro-nutrients [16]. Because of poor absorption, they are retained in the intestine for longer time where they can promote beneficial effect, namely by affecting the GM community [17]. The impact of dietary polyphenols on gut ecology and the mechanism underlying the putative beneficial effects on GI and extra-intestinal diseases have been depicted during the last decade [18]. In this review, we firstly overview the main features and functions of GM on human health, then we summarize the links between GM dysbiosis and diseases (intestinal and extra-intestinal) and, finally, we critically review the evidences available concerning the impact of polyphenols in GM based on preclinical and clinical data.

## 2. Gut Microbiota Dysbiosis and Disease

### 2.1. Gut Morphology and Healthy Microbiota Composition, Diversity and Functions

The human GI tract, with 250–400 m^2^ surface area, is one of the chief associations between the host body, external environmental factors and internal antigens. During average lifespan, about sixty tonnes of food and a huge number of external microbes traverses the GI tract, jeopardizing gut integrity. The set of gut microbes- bacteria, archaea, viruses (mainly phages), eukaryotes (mainly yeasts), and other microbial species-, collectively referred as gut microbiota, has jointly developed with the host during millions of years, forming a complex and symbiotic connection [19].

Among the large population of microbial species, about 10^14^ cells inhabit the human GI tract. The composition of GM varies along the GI tract in accordance with the morphological and physiological features of digestive system region. The concentration considerably rises from the proximal to the distal gut, together with an enrichment in anaerobes [1,2]. GM composition in the initial proximal portion of the small intestine, predominantly in the duodenum, is identical to that of stomach, given the acidic conditions that results from the chyme of the stomach and biliary and pancreatic secretions. The diversity and number of bacteria increases in the distal portion, from duodenum to ileum, accompanying the gradual increase of pH. In this portion, predominate *Lactobacillus* and *Clostridium* species of the Firmicutes phyla, *Escherichia coli* of the Proteobacteria phyla, as well as Bacteroidetes and gram-negative facultative anaerobes [1,2]. The conditions in large intestine, namely in the colon, with a more beneficial nutritional milieu and pH (5.7–6.8), favor bacterial growth, allowing a more diverse, dense and complex microbial community, essentially composed of obligate anaerobes that survive at low concentrations of oxygen. In the colon, the predominant bacteria are *Ruminococcus*, *Lactobacillus* and *Clostridium* species of the Firmicutes phyla, and *Bacteroides* and *Prevotella* of the Bacteroidetes phyla. There are other phyla present in the adult GM, thought in lower abundance, including Actinobacteria, Fusobacteria, Proteobacteria and Verrucomicrobia, as well as some facultative anaerobic bacteria [20,21]. Bacterial composition and functions also varies between the intestinal lumen and the mucus layer of the intestinal mucosa, with a different proportion of anaerobic and aerobic species [1,2,22].

Several recent pieces of evidence support the idea that, against what was previously thought, the microbial colonization of humans starts in utero from maternal commensal microbes, as suggested by the bacteria found in the amniotic fluid, maternal placenta, umbilical cord blood and in meconium (the first “faeces-like excretion”) [23,24,25].

GM phylogenetic diversity increases with growth and development of the host; around 2–3 years age, a complex and stable community of microorganisms is formed. Based on different methods of molecular profiling, it is predictable that about one thousand species of bacteria inhabit the gut, mainly anaerobes, including Firmicutes, Bacteroidetes, Actinobacteria, and Proteobacteria. However, the two predominantly phyla Bacteroidetes and Firmicutes constitute over 90% of all bacterial species in the intestine [26,27].

The healthy GM is stable and contributes to several important physiological host functions (Figure 1). In brief, GM exerts important (i) metabolic effects (co-metabolism), such as synthesis of vitamins and fermentation of carbohydrates, lipids, and proteins; (ii) structural defending properties against pathobionts by preserving integrity and regulating the permeability of gut barrier, thus contributing to host homeostasis; as well as (iii) “education” of host immunity [5]. The intestinal bacteria have ability to synthesize essential and nonessential amino acids. They can produce numerous vitamins and short-chain fatty acids (SCFAs) and are able to carry out biotransformation of bile. Moreover, GM can metabolize some complex oligosaccharides which escaped the digestion, namely several barely digestible polysaccharides, including cellulose, hemicellulose, resistant starches, gums and pectins, unabsorbed sugars and alcohols obtained from the diet. This helps in the retrieval of absorbable substances to the host and in turn the bacteria derive nutrients and energy for their growth and multiplication [5,28]. GM plays a vital role in host immune system development and maintenance. Many findings proved the crucial role of GM in the regulation of antigen presenting cells (APCs) development, namely B-cells, macrophages and dendritic cells, which have capability to protect the body from pathogens while being immuno-tolerant towards gut microbes [29]. Studies with germ-free mice have reported that symbiotic intestinal bacteria are indispensable for the growth and functioning of intestine-linked lymphoid tissues (namely Peyer’s patches and mesenteric lymph nodes) and specific lymphocytes [30]. Under normal conditions, GM/immune system interaction stimulates the generation of beneficial responses towards pathogens and the protection of the regulatory pathways associated with the tolerance preservation towards safe antigens. In the developed countries, factors like unwarranted usage of antibiotics and major changes of dietary habits might have influenced the selection of a microbial community composition that fails to create balanced immune responses. This could be ascribed towards the noteworthy increase in autoimmune and inflammatory disorders spanning the last few decades [31].

### 2.2. Factors Responsible for Gut Microbiota Alteration (Dysbiosis)

In the mid-20th century, Metchnikoff first coined the term “dysbiosis” to depict the changes in intestinal bacteria, suggesting a link with immune homeostasis impairment and development of intestinal disorders. In general, dysbiosis can be classified as (1) a decrease in number of symbionts; (2) an unwarranted growth of pathobionts; and (3) a loss of diversity. It has been reported that these 3 different types can co-exist, which is most often the case. Several factors are responsible for an impaired GM composition and/or function-dysbiosis-, including age, diet and lack of exercise, stress, drugs and xenobiotics [1,4,5].

Major GM alterations have been reported in early phase of life, particularly in the first years, when there is a marked increase in numbers and diversity. The maturation and evolution of the human gut microbes is an example of ecological succession [32,33]. After an initial stage of massive new colonization, when the microbial composition is highly variable between individuals, GM undergo successive changes in composition and function until a stable climax community is established. However, major differences have been reported between adult populations of distinct geographic World regions, which might be explained by a diversity of factors, including both genetic and environmental, particularly dietary patterns, hygienic conditions, as well as use of antibiotics and other drugs [34,35]. Furthermore, aging also affects GM composition, changing the number and particularly the diversity of bacteria, with a decrease in the *Firmicutes* to *Bacteroides* (F/B) ratio, which might be partially explained by modifications caused by an altered immune-inflammatory milieu–the so called immunosenescence [10]. Further causes of aging-related altered GM are the deterioration of physical health, including loss of dentition, impaired salivary function, digestion and transit time in the GI tract, as well as abnormal dietary nutrients resulting in malnutrition [36,37].

Diet is in fact a chief GM modulator, affecting both the composition and functions, thus contributing to maintain health or to favor disease states [38]. For instance, breast milk contains certain oligosaccharides that cause proliferation of *Lactobacillus* and *Bifidobacterium* that are predominant in the infant gut and could contribute to immune system development. Similarly, demographic dietary factors result in differential variation in the GM [39]. Rural Africa children, usually consuming plant polysaccharides, had lower faecal levels of *Firmicutes* and higher of *Bacteroidetes* (particularly *Prevotella* and *Xylanibacter*) when compared with Italian kids that present high amounts of Enterobacteriaceae (especially *Shigella* and *Escherichia*). *Prevotella* and *Xylanibacter*, in particular, can cause an increment of beneficial SCFAs as a result of cellulose and xylans degradation, suggesting an adaptation to take full advantage of energy extraction from fibre-rich diet. In fact, resistant starch in human diets promote increment of *Ruminococcus bromii* and *Eubacterium rectale* in faeces, which show a relationship with fibre fermentation [40]. Several evidences from preclinical models and from humans studies consistently show that high-fat and/or high-sugar diets modifies the GM profile towards dysbiosis, while vegan diet, prebiotics (such as inulin) and/or probiotics causes beneficial effects on GM composition and function, accompanied by reduced adiposity, inflammatory molecules, including lipopolysaccharide (LPS), and other positive effects on metabolic hemostasis [4].

Some recent evidence points to the impact of artificial sweeteners and emulsifiers in GM. Artificial sweeteners, frequently used as sugar alternatives, have been “generally recognized as safe” (GRAS) by regulatory agencies. However, investigations have reported that some of them, including aspartame, sucralose and saccharin, may upset the balance and diversity of GM. In fact, rats orally treated with sucralose for 84 days showed increased gut levels of Clostridia, Bacteroides, and total aerobic bacteria, upregulated expression of bacterial pro-inflammatory genes, as well as disturbed faecal metabolites and augmented faecal pH [41]. Emulsifiers, which are food additives often present in processed food, have also been associated with altered GM in animals. Mice treated with carboxymethylcellulose and polysorbate-80, two commonly used emulsifiers, revealed decreased numbers of Bacteroidales and Verrucomicrobia together with increased amounts of Proteobacteria, which usually is linked with mucosal inflammation [42]. Altogether the previous examples reinforce the important role of diet in modulation of bacterial-derived metabolites than just influencing the microbiota community for short-term period [43].

Several non-dietary environmental factors are able to impact the human GM towards dysbiosis, including stress, smoking habits and lack of exercise practice, which might be due to, among other factors, the associated pro-oxidative and pro-inflammatory milieu [10]. In addition, medication is a major modulator of the gut ecology. By definition, antibiotics alter GM composition and functions [44,45] and the loss of colonization resistance is an earlier effect of antibiotics on the gut. This loss resulted in much easier rate of colonization by *Salmonella* following antibiotic treatment. Recent studies in mice reveal that antibiotics cause an increment of gut free sialic acid derived from the host, that can be further used by opportunistic pathogens to growth (namely by *Salmonella typhimurium* and *Clostridium difficile*) [46].

## 3. Linkage of Dysbiosis to Diseases

**Figure 2:**
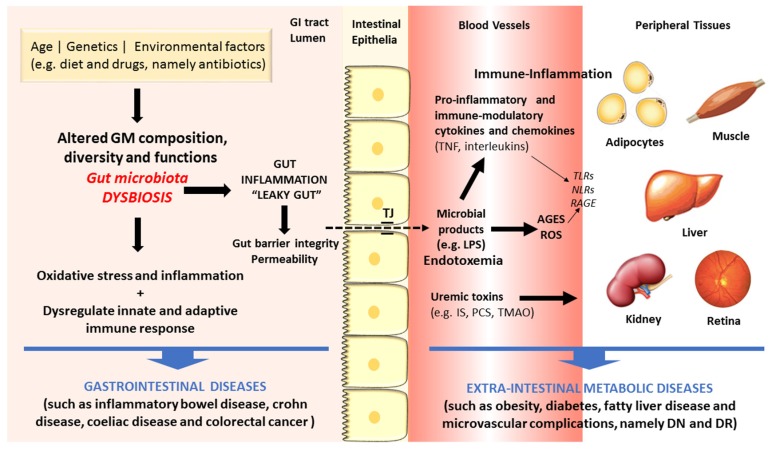
Schematic diagram of some of the main factors and pathways linking dysbiosis to gastrointestinal and extra-intestinal metabolic and vascular diseases.

There are increasing evidences in favor of an association between dysbiosis and diseases, including those of the GI tract, such as IBD, UC, CD and CRC, but also some extra-intestinal metabolic disorders, such as obesity, diabetes and macro- and microvascular complications [8,9,10,11]. Figure 2 summarizes some of the main linkages of dysbiosis to GI and extra-intestinal metabolic and vascular diseases.

The gut microbes act in tandem with the defense and immune systems of the host to protect against colonization and invasion of pathogens [21]. Recently, a remarkable amount of experimental data has firmly advocated the important role of GM in human health and disease through several molecular mechanisms. First, the gut microbes have the capacity to increase energy and nutrient extraction from the diet, and it can also alter appetite signaling; second, the human GI flora also offers a physical barrier to protect the host against pathogenic microorganisms, inhibiting their colonization by producing antimicrobial compounds [20,47,48,49]. During metabolism of xenobiotics, the host and its intestinal microbes generates molecules that play crucial part in exchanging information among host cells and its microbial symbionts. Furthermore, there are relevant uremic toxins derived from the intestinal microbial metabolism of proteins, amino acids and other metabolites, including mainly phenols and indoles bounded to proteins and products of metabolism of phenylalanine and tyrosine, such as p-cresol (PC) and p-cresyl sulfate (PCS), and of tryptophan degradation, such as indoxyl sulfate (IS) and indoleacetic acid [50]. Finally, amines and polyamines are also originated by GM metabolism. A major example is choline, a crucial nutrient for lipid metabolism that is metabolized into toxic trimethylamine and further converted to trimethylamine-*N*-oxide (TMAO) in the liver, which is a promoter of cardiovascular and renal diseases [51,52].

Changed GM composition have been reported in IBDs subjects when compared to controls, though no uniform pattern of alterations has yet been observed [53,54]. The mechanisms underlying the association between GM dysbiosis and evolution of IBDs include the pro-inflammatory and pro-oxidative profile generated in the intestinal lumen and adjacent layers [55,56]. In addition, debates are also going on regarding the crucial role of heat shock proteins (HSPs) in the pathogenesis of IBD, namely due to their participation in several relevant biochemical pathways, such as folding, translocation, and ubiquitinylation of intracellular proteins, as well as due to their capacity to excite innate and adaptive immune response, thus serving as primary autoimmune response targets [57]. The Th17/Treg cells balance, characterized by pro-inflammatory and anti-inflammatory cytokines, which is pivotal for the host’s intestinal homeostasis and induction or inhibition of colonic inflammation, is highly influenced by GM composition. Under inflammatory conditions, including IBD and other GI diseases, antigens derived from a dysbiotic GM activate immune cells (namely Th1 and Th17), causing tissue damage, reduction of mucus layer, and exacerbated penetration of microbes in the intestinal tissues. Consequently, there is an augmented uptake of microbial antigens and toll-like receptor (TLR) ligands that perpetuate the immune responses [8,58].

As above referred, the composition of intestinal microflora is affected by several factors, including lifestyle habits, particularly diet and exercise, which are risk factors for cardiometabolic diseases, such as obesity and T2DM. These conditions have been viewed as the result of an intricate crosstalk between individual genetics, environment influences, including the dietary pattern, as well as the intestinal microflora [59]. Increasing evidences coming from preclinical and clinical studies support the existence of a dysbiotic GM in obesity and T2DM, characterized by lower diversity and resilience [60]. The putative association between dysbiosis and the development of obesity, T2DM and its serious vascular complications has been suggested based on several distinct mechanisms (Figure 2). In brief, GM dysbiosis is a trigger for gut barrier integrity breakdown, with changes on the expression of tight proteins, followed by augmented permeability and consequent translocation from the gut lumen to the bloodstream of bacteria fragments, namely lipopolysaccharide (LPS) and peptidoglycan (PG), and uremic toxins, thus inducing endotoxemia, also referred as a low-grade inflammation state [61,62]. The so-called microbe-associated molecular pattern (MAMP) cause a pro-inflammatory response by binding to toll-like receptors (TLRs), namely TLR4, which evokes a cascade of responses that culminate in pro-inflammatory molecules’ release, which will then affect glucose and insulin metabolism and/or signaling [63]. This is further fueled by advanced glycation end products (AGEs) and other oxidative pathways which are deeply involved in the metabolic impairment in obesity and diabetes development. Concomitantly, several evidences strongly suggest that signals coming from the dysbiotic GM modulate immunometabolism by interfering with epithelial and immune cells, generating an immune-inflammatory milieu that favors the progression of diabetes and its complication [10]. In fact, diabetic gut dysbiosis seems to contribute not only to obesity and diabetes development but also to the progression of some of its main microvascular complications, including diabetic retinopathy (DR) and nephropathy (DN). It is suggested that an increase of circulating bacterial endotoxins, particularly LPS, might play a major role in the low-grade inflammation typical of obesity, diabetes and microvascular complications. The translocation of bacterial components and other microbial-derived products through the impaired intestinal barrier to the systemic circulation can contribute to the pro-inflammatory and pro-oxidative profile found in DN and DR, as well as to the overactivated innate and adaptive immunity [10]. These mechanisms, overall, can be pivotal to the progression of metabolic and vascular complications of diabetes (Figure 2).

The question remains whether dysbiosis is directly linked to metabolic disorders, particularly obesity, T2DM and its vascular complications, or whether the impaired GM composition is an adaptation to alteration of host’s diet and other modulatory factors. There following findings might help to achieve valid answers: (i) microbiota transfer from lean donors into subjects with metabolic disease causes amelioration of insulin sensitivity and (ii) change in human’s diet patterns promote a quick and reversible alteration in the composition of dominant GM members [64,65].

## 4. Effects of Polyphenols on Gut Microbiota

### 4.1. Major Classes of Polyphenols

Dietary polyphenols are compounds of natural origin present in food items such as vegetables, fruits, cereals, tea, coffee, dark chocolate, cocoa powder, and wine. Chemically, these compounds constitute a big heterogeneous collection of compounds, but with structural units common in all phenolic compounds (hydroxylated aromatic rings or phenol rings) [13,66]. According to the number of phenol rings that they contain and the structural elements binding these rings, the phenolic compounds are divided in groups. The main groups of dietary polyphenols are: phenolic acids, flavonoids, tannins, stilbenes and diferuloylmethanes [13,66].

**Figure 3:**
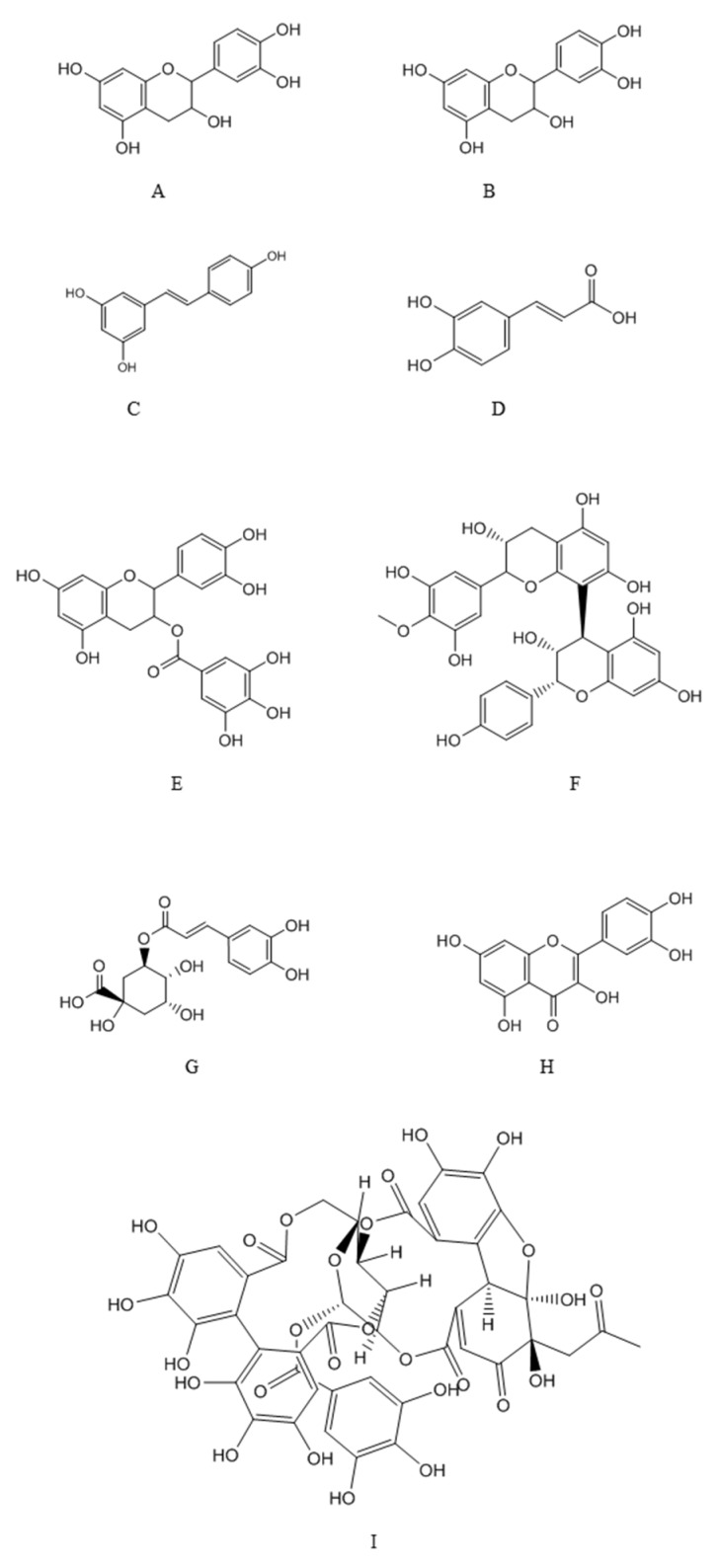
Structure of main polyphenols classes. A: catechins; B: Epicathechins; C: Resveratrol; D: Caffeic acid; E: Proanthocyanidin; F: Epicatechin gallate; G: chlorogenic acid; H: Quercetin; I: Ellagitannin.

Figure 3 shows the chemical structures of some of the principal classes of dietary polyphenols. Phenolic acids are compounded by a phenol ring and a carboxylic function and are divided in benzoic acid derivatives (gallic and protocatechuic acids) and cinnamic acid derivatives (p-coumaric, caffeic and ferulic acids). Stilbenes are hydrocarbons with a trans-ethene double bond replaced by a phenyl group on both carbon atoms of the double bond [67]. They appear in low percentage in plants, being resveratrol and its isomer *trans*-resveratrol the most abundant, usually found in grapes and wine. Flavonoids comprise more than 10,000 natural compounds, being nearly ubiquitous in plants (more than 9000 species) [13,66,67]. These compounds have a huge structural diversity, but are derived from a common biosynthetic pathway, the phenylpropanoid metabolic pathway, which incorporates precursors from the shikimic acid and acetate-malonate pathways, lead; well-known examples are kaempferol, quercetin, luteolin, (epi)catechin, (epi)gallocatechin, etc. Tannins are polyphenolic compounds that precipitate proteins, in especially salivary proteins, giving them an astringent character. This property explains their protective role in plants against pathogens and herbivores. They are water-soluble polyphenols with high molecular weight (500–3000 Da), classically divided in condensed tannins or proanthocyanidins (polymers of flavan-3-ol units) and hydrolysable tannins (esters of phenolic acids and a cyclic polyalcohol, usually glucose). Diferuloylmethanes are a small group of phenolic compounds; a well-known compound belonging to this class is curcumin, whose major source is the spice turmeric [66].

### 4.2. Interplay between Polyphenols and GM and Impact on Disease

**Figure 4:**
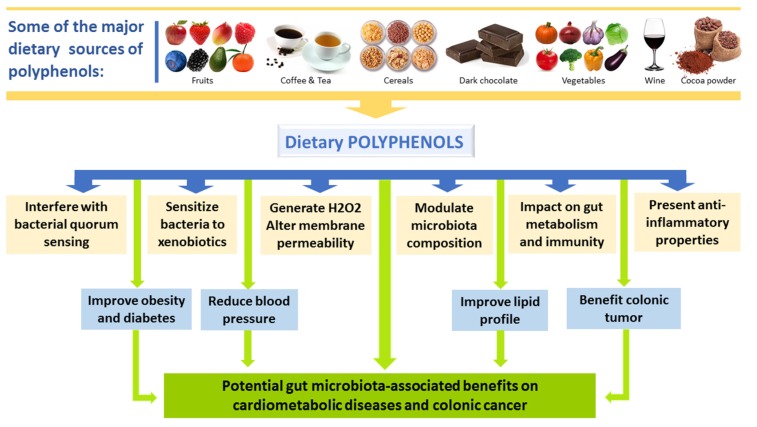
Major sources of dietary polyphenols and the potential gut microbiota-associated benefits on human health.

Once consumed, dietary polyphenols are apparently perceived as xenobiotics in humans and their biological availability is reasonably poor as compared with micro- and macro-nutrients. Moreover, structural complexity and polymerization also affect their absorption in small intestine [68]. Absorption of the ingested polyphenol in the small intestine is very low (about 5–10%). The left over polyphenols (90–95%) may accumulate up to the millimolar range in the large intestine along with the bile conjugates released into the lumen and are exposed to the gut microbial enzymatic activities [69]. Recent studies support that dietary phenolic substances reaching the gut microbes, as well as the aromatic metabolites generated, may modify and produce variations in the microflora community by exhibiting prebiotic effects and antimicrobial action against pathogenic intestinal microflora [18,70,71]. Figure 4 summarizes the major sources of dietary polyphenols and the potential gut microbiota-associated benefits on human health.

In humans, the metabolic destiny of dietary polyphenols is a relevant issue that deserves consideration. The small intestine is responsible for the absorption of a low amount of dietary polyphenols, mostly after de-conjugation reactions like de-glycosylation [72]. After absorption into the small intestine, the polyphenolic compounds having lesser complexity may pass through biotransformation in the enterocytes and then in the hepatocytes via Phase I (oxidation, reduction and hydrolysis) and especially Phase II (conjugation) reactions. These transformations produce a chain of water-soluble conjugated metabolites (glucuronide, sulfate and methyl derivatives) which are readily released in the systemic circulation for subsequent delivery to organs and excretion by the urine. Polyphenolic backbone of the 90–95% unabsorbed polyphenols is acted upon by the colonic bacterial enzymes in the large intestine, and consecutively generate metabolites having diverse physiological implications [73].

Colonic microflora may transform the polyphenols into bioactive compounds, which have the ability to influence the intestinal ecology and affect human health. Studies in animals and in humans have shown that prescribed amounts of particular polyphenolic compound may amend the gut microflora composition resulting in inhibition of certain bacterial groups, while others can flourish in the available niche of the ecosystem. Table 1 summarizes the main studies concerning the influence of polyphenols on GM.

An in vitro study reported that flavan-3-ol monomers, namely, (+)catechin and (−)epicatechin, may have ability of impelling the bacterial population in large intestine [86]. (+)Catechins considerably subdued the growth of *Clostridium histolyticum* and boosted the growth and development of members of the *Clostridium coccoides*-*Eubacterium rectale* group and *E. coli*, while growth of *Lactobacillus* spp. and *Bifidobacterium* spp. remained comparatively unaltered. Proanthocyanidin-rich red wine extract has been shown to swing the preponderance of *Bacteroides*, *Propionibacterium* and *Clostridium* spp. towards the predominance of *Bacteroides*, *Bifidobacterium* and *Lactobacillus* spp. in a colon cancer animal model [75]. In another study, resveratrol from grape stimulated faecal cell counts of *Lactobacillus* and *Bifidobacterium* spp. in the rodent model of colitis induced by dextran sulfate sodium (DSS) [80].

Mechanisms of action of dietary polyphenols varies in Gram positive and Gram-negative bacteria due to changes in cell membrane structure. Polyphenols have ability to bind bacterial cell membranes in a concentration dependent manner, therefore altering functional aspects of membrane and thus preventing their growth. Catechins, interact with many bacteria (*Bordetella bronchiseptica, E. coli, Klebsiella pneumonie, Serratia marcescens, Pseudomonas aeruginosa, Salmonella choleraesis, Bacillus subtilis* and *Staphylococcus aureus*) by producing H_2_O_2_, by changing the microbial cell membrane permeability, as well as by sensitizing bacteria to the effects of antibiotics, as was found with epicatechin gallate in methicillin-resistant *S. aureus* treated with beta-lactam antibiotics [74].

Antimicrobial phenolic rich extracts from dietary spices and medicinal herbal samples (Padang cassia, Chinese cassia, oregano, Japanese knotweed, pomegranate peel and clove) showed ability to control five food-borne pathogenic bacteria (*Bacillus cereus*, *Escherichia coli*, *Salmonella enterica subsp. enterica serovar typhimurium*, *Shigella flexneri* and *Staphylococcus aureus*). The probiotic effects were also examined on five lactic acid bacteria *(Lactobacillus acidophilus*, *L. delbrueckii subsp. bulgaricus*, *L. casei*, *L. plantarum* and *L. rhamnosus).* The results demonstrated a co-existence with lactic acid probiotic bacteria and none of the edible plant extracts showed inhibitory growth effect except on *L. bulgaricus*. A possible explanation for these results is that lactic acid bacteria survive in a relatively low pH environment, producing organic acids during fermentation and detoxifying the phenolic acids through metabolism [81].

Polyphenolics can also hinder with bacterial quorum sensing, that is accomplished by generating, liberating and sensing small signal molecules recognized as auto inducers (oligopeptides in Gram-positive bacteria and acylated homoserine lactones in Gram-negative bacteria) [92]. Green tea and red wine polyphenols powerfully hinder the key toxin of *Helicobacter pylori* which is known as the VacA toxin [75]. The inhibitory actions of food polyphenols against *H. pylori* may comprise inhibition of urease activity, influencing multiplication of bacteria and destroying bacterial cell membrane integrity, hence causing bacteria to become extra sensitive to xenobiotics like antibiotics and causing collapse of proton motive force via loss of H^+^ ATPase and membrane-associated tasks [93]. Flavanoid B ring may take part in intercalation or H-bonding with nucleic acid base pair stacking, and this could elucidate the inhibitory action of flavonoids on DNA and RNA biosynthesis [66,94]. Binding of quercetin to *E. coli* DNA gyrase (GyrB subunit) has been reported to produce the enzyme’s ATPase activity [95]. Many mechanisms of polyphenols’ action on gut microbiota functions are still not known and additional research efforts are solicited for proper understanding.

Wang et al., investigated the GI protective effects of polyphenols from bee products: Prunella vulgaris honey (PVH) [84] and Chinese and Brazilian propolis [83]. PVH significantly modulated the GM composition in the DDS-induced colitic rats, increasing the Bacteroidetes/Firmicutes ratio and restoring *Lactobacillus* spp. populations. Similar results were obtained for polyphenols from propolis that significantly reduced the Bacteroides spp. Also mushrooms rich in antioxidant polyphenols compounds are able to modulate GM composition. In particular, *Ganoderma lucidum* mushrooms showed capacity to reduce the Firmicutes/Bacteroidetes ratios and endotoxin-bearing Proteobacteria levels in the DSS-induced colitis model. In addition, the intestinal barrier integrity was reinforced and endotoxemia attenuated, together with beneficial effects on body weight, inflammation, and insulin resistance [85]. During human intervention study, flavonols were reported to stimulate growth and proliferation of *Bifidobacterium* spp. and *Lactobacillus* spp. which might have been partially accountable for the perceived decline in the concentration of plasma C-reactive protein (CRP), an inflammatory blood biomarker and a hallmark of the acute phase inflammatory response [71]. Likewise, in an in vitro model bacterial fermentation of water-insoluble cocoa fractions was related with rise in *Lactobacilli* and *Bifidobacteria* along with butyrate generation; in addition, alterations in these microbes were linked with substantial decline in plasma trilglycerides and CRP, signifying the prospective benefits associated with the inclusion of flavonol-rich foods in diet [96]. Consumption of red wine polyphenols on regular basis caused noteworthy reductions in the blood pressure, as well, as in plasma triglycerides and HDL-cholesterol levels. The reduction of such parameters might be partially attributable to the polyphenol-mediated induction in the proliferation of *Bacteroides* spp. [88]. Furthermore, consumption of red wine polyphenols led to a noteworthy decline in uric acid levels. It can be accounted for by the substantial enhancement in *Proteobacteria* population noticed in this stage, which break down uric acid [97]. The weight-reduction activity of green tea, fruits and vinegar wine in obese individuals may be partially associated with their polyphenol contents that modifies the gut microflora either by the glycan-degrading ability of *Bacteroides*, that is higher than *Firmicutes*, or by the metabolic end products resulting from colonic metabolism of polyphenols [89]. Monagas et al. reported that dihydroxylated phenolic acids (3-hydroxyphenylpropionic acid, 3,4-dihydroxyphenylpropionic acid and 3,4-dihydroxyphenylacetic acid) produced from the microbial transformation of proanthocyanidins exhibited potent in vitro anti-inflammatory activities, plummeting the secretion of cytokines namely, TNF-α, IL-1β and IL-6 in lipopolysaccharide-induced peripheral blood mononuclear cells from normal individuals [90]. Microbial metabolites of phyto-polyphenols have been shown to decrease the risk in the metabolic syndrome. During a study on in vitro model of protein glycation, Verzelloni et al. revealed that pyrogallol and urolithins, the two microbial metabolites obtained from ellagitannin are highly anti-glycative in comparison to parent polyphenolic compounds. Moreover, protein glycation has been reported to play a vital pathological role in diabetes and associated complications, including blindness [98].

In a randomized, single blind, crossover study, Zanzer et al. (2017), reported that polyphenols from spice like turmeric (curcumin, demethoxycurcumin and bisdemethocycurcumin), star anise (quercetin derivatives, kaempferol derivatives and isorhamnetin derivatives), ginger (gingerols and shogaols) and cinnamon (procyanidins, cinnamic acid, kaempferitrin, cinnamaldehyde and 2-hydroxycinnamaldehyde), lowered cardiometabolic risk acting on the gut through glucose uptake inhibition and appetite modulation [91]. Four standardized beverages (220 mL corresponding to 185 mg of gallic acid equivalents) from flavored water and extract of turmeric (*Curcuma longa*), star anise (*Illicium verum* L.), ginger (*Zingiber officinale*) or cinnamon (*Cinnamomum burmannii*) were administered at eighteen (11 men and 7 women) randomized volunteers. Interesting results were obtained for turmeric and cynnamon that, before intake of a carbohydrate challenge, reduced the postprandial blood glucose phase without affecting insulin; turmeric reduced the ‘desire to eat’ and ‘prospective consumption’, and increased the postprandial levels of the gut hormone PYY (peptide tyrosine-tyrosine) [91].

Another in vivo study was conducted with polyphenols from green algae in high-fat/high-sucrose diet and streptozotocin-induced diabetic mice [82]. An ethanolic extract of *Enteromorpha prolifera* passed through an ultrafiltration membrane of 3 kDa (EPE3K) was able to repair inflammation of hepatocytes caused by diabetes and to improve the liver cells in T2DM mice. EPE3k treatment had a strong hypoglycemic activity and improved the oral glucose tolerance on streptozotocin-induced diabetic mice. In addition, a decrease of body weight of mice and a hypoglycemic activity were observed, together with a beneficial impact on GM, with a decrease of *Turicibacter* and *Akkermansia* and an increase of *Alistipes* [82].

Several studies have revealed the association between the microbial metabolites of dietary polyphenols and cancer prevention. Results of these studies have demonstrated phylum level variations in the gut microflora of patients with- and without-colorectal cancer. However, some phyla are augmented and others are diminished, but precisely how these modifications influence the cancer progression is not clearly known [99]. Some dietary polyphenolic compounds may also modulate bacterial metabolic enzymes, consequently affecting the risk in cancer patients. For instance, rats fed daily with resveratrol intragastrically (8 mg/kg body weight) considerably decreased the activities of faecal and host colonic mucosal enzymes, viz., α-glucuronidase, nitroreductase, β-galactosidase, mucinase, and α- glucosidase, as compared to control animals (21%, 26%, 37%, 41% and 45%, respectively). The reduction in bacterial enzyme activity was linked with a major decline in colonic tumor occurrence in the resveratrol-supplemented rats as compared with normal control rats, but it is not clear whether these changes resulted from alterations of enzymatic activity within a subpopulation of microflora or a variation in the proportion of particular gut microflora [79]. The stilbene resveratrol has been shown to negatively affect the progression of colon cancer. The resveratrol exhibits anti-inflammatory activity by inhibiting proinflammatory mediators, modulating eicosanoid biosynthesis and inhibiting enzymes including COX-2 (cyclooxygenase-2), IL6 (interleukin 6), TNF-α (tumor necrosis factor-alpha), AP-1 (Activator protein 1), NF-kB (nuclear factor kappa-light-chain-enhancer of activated B cells), and VEGF (vascular endothelial growth factor) [100]. In an in vitro study, COX-2 activity was inhibited by numerous phenolic compounds probably by binding to the enzyme [101]. In mice model, coffee and caffeic acid are reported to inhibit colon cancer metastasis and neoplastic cell transformation by inhibiting TOPK (T-LAK cell-originated protein kinase) and MEK1 [78]. Cardona and co-workers have investigated the influence of certain intestinal polyphenolic metabolites (3,4-dihydroxyphenylacetic acid, 3-(3,4-dihydroxyphenyl)-propionic acid, metabolites of chlorogenic acid/caffeic acid and quercetin) on variation in enzyme activities associated with inflammation and detoxification in LT97 human adenoma cells. They reported upregulation of GSTT2 and a down-regulation of COX-2 which could possibly add to the chemopreventive potential of polyphenols after their metabolic breakdown in the intestine [69].

Overall, dietary polyphenols have shown, both in preclinical and in clinical studies, several benefits on distinct disorders due to effects on GM, although further experimental evidences are still warranted to elucidate the precise molecular mechanisms involved.

## 5. Strategies to Improve Efficiency of Pre- and Probiotics Delivery

As mentioned above, the low bioavailability represents polyphenols’ major drawback, which compromises the possible health benefits. This is also true for probiotics since some bacteria cannot freely circulate through the GI tract without being killed. In order to overcome this limitation, among different therapeutic approaches, many strategies have been recently proposed. They are focused on the possibility to allow targeted local delivery to the intestinal region, thus reducing the systemic diffusion, improving the effectiveness of delivering on the appropriate level of the GI tract, which is frequently, and consequently, associated with the occurrence of less side effects [102,103,104]. Different strategies have been proposed exploiting physiological changes in the intestinal tract, such as pH-sensitive delivery systems, enzyme linkers, pressure-dependent delivery systems, osmotic controlled and prodrugs [105]. In particular, polymers such as Eudragit^®^, Poly (methacrylic acid-co-ethyl acrylate), cellulose acetate phthalate (CAP)), hydroxyl propyl methylcellulose phthalate (HPMCP), have been widely used for coating capsules and tablets [106]. The peculiar properties of micro- and nanoparticles such as size, shape, surface, stability (4S) [107], strongly influence the in vitro and in vivo fate of active compounds, making these carriers a potential valid strategy also for local intestinal region. To improve the polyphenols activity and the intestinal or colon release, different flavonoids spray-dried microcapsule obtained by cellulose derivative with gastro-resistant swelling and controlled release properties were formulated. Satisfying data were obtained at pH 1.0 (USP gastric simulated fluid) with a release <20%, associated to a complete release of glycosides naringin and rutin at pH 7.0 (USP intestinal simulated fluid). Complete intestinal release of their aglycones was obtained adding surfactants without altering the gastric release [108]. Chung et al. (2014) used two different types of HPMCP (S and L) to coat green tea catechins to improve their digestive stability and intestinal transport [109]. The bioavailability was investigated on in vitro gastrointestinal model coupled with Caco-2 cells. The intestinal transport rate of L-HPMCP showed an increase of 3.47 times in respect to green tea catechins alone and resulted better than S-HPMCP (increase of 1.50 times). In addition, the digestive stability was improved (2.74 times for S-HPMCP and 3.56 times L-HPMCP). Eudragit S100 (ERS100) was used also in presence of PLGA in 2:1 weight ratio to obtain curcumin drug delivery microparticles as anti-inflammatory agents in colitis tissues. The carrier showed high curcumin loading, well-controlled timed release for more than 24 h and possessed a better efficacy on ulcerative colitis with respect to the pure curcumin [110]. To avoid the resveratrol intestinal release, other matrices such as silk fibroin nanoparticles were developed. The intestinal anti-inflammatory was administered intracolonically and evaluated in an experimental model of rat colitis. The resveratrol fibroin nanoparticles showed a high efficacy decreasing the myeloperoxidase activity and inhibiting the expression of TNF-α, IL-1, IL-6 and IL-12. Thus, this approach seems to be an attractive strategy for the controlled release of resveratrol to intestinal inflammation [111]. An interesting approach was the reported by Shinde et al. (2014) that investigated the effects of alginate microencapsulation on synergy between polyphenols apple skin extract (ASPE) and the probiotic bacteria (PB), *Lactobacillus acidophilus.* [112]. In particular, the survival and growth of PB alone and formulated was analyzed after 50 days of storage at 4 °C in milk; 0.5% of alginate solution was employed to produce PP-core and PP-PB core solution for 1% alginate, obtained by co-extrusion method. Studies on the antioxidant activity showed a high value for the aqueous PB–aqueous PPs–alginate bead system, due to the great efficacy of ASPE in combating the oxidative stress. Instead, in the presence of ASPE, the PB cell death rate decreased and the *Lactobacillus acidophilus shelf life* increased. This suggests that the formulation of PB in the presence of ASPE can allow a PB release in a dose higher than the minimal requirement of 10^−6^–10^−7^ CFU, protecting it and offering an approach for benefit also from the antioxidant health effects of PPs. Another probiotic, the yeast *Saccharomyces cerevisiae boulardii*, was microencapsulated in an alginate-inulin-xanthan gum mixture [113]. There was an increased ability to survive and growth in berry juice for 4 weeks storage at 4 °C. In addition, the microcapsules absorbed different polyphenols and anthocyanins, protecting also them from harsh conditions of the GI environment. This behavior add value to this probiotic formulation due to polyphenols and anthocyanins beneficial effects on microflora and human health. In fact, Fleschhut et al. (2006) demonstrated that the anthocyanins were degraded by intestinal microflora into phenolic acids [114]. They are responsible for the health benefits of dietary anthocyanins [115] which are associated also to a beneficial modulation of the GM, particularly increasing the *Bifidobacterium strains* [116].

Based on the increasing understanding of GM and its possible targeting, continuous efforts are being developed by researchers all over the world, with the contribution of bioengineers, cell biologists, material chemists, and pharmaceutical technological scientists. A deeper understanding of the roles of polyphenols that can act in synergy with different probiotic strains in modulating GM composition and function, together with epigenetics and metabolomics findings, would allow the development of innovative approaches to prevent and manage targeted microbiota diseases contributing to achieving the goal of personalized drug delivery, nutraceuticals or functional food.

## 6. Conclusions and Perspectives

GM plays a central role in many mechanisms crucial for host physiology and metabolism. Multiple factors, including unhealthy dietary habits, can cause disruption of microbiota equilibrium (dysbiosis), which has been associated with gastrointestinal diseases (namely IBD, among others) as well as with extra-intestinal metabolic disorders, namely obesity and diabetes. Polyphenols, highly present in a wide range of healthy foods (namely vegetables and fruits), have been linked with beneficial effects on multiple disorders, including cardiometabolic, neurodegenerative and oncologic, which might be due to its antioxidant, anti-inflammatory and other cytoprotective properties. Evidence from preclinical and clinical studies suggest that polyphenols are able to express prebiotic properties and exert antimicrobial activities against pathogenic gut microflora. Although the precise mechanisms deserve further clarification, dietary polyphenols have shown benefits in distinct disorders, accompanied by a major impact on GM towards symbiosis. Unfortunately, the therapeutic/nutraceutical use of polyphenols has been seriously compromised by the lower bioavailability and inability to efficiently achieve the targets (tissues/cells/gut bacteria). In order to overcome this limitation, during the last years several approaches have been developed, aiming to transport polyphenols throughout the GI tract and deliver the phenolic compounds in the targeted intestinal regions. In addition, probiotic inserted in flavonoids formulations can better reach the target regions and act in a synergic fashion, improving efficiency. Biotechnological advances achieved during recent years have paved the way to efficient use of phenolic compounds targeting GM in a broad range of disorders characterized by a dysbiotic phenotype.

## Figures and Tables

**Table 1:** Main preclinical and human data reporting the effects of polyphenols on gut microbiota and associated mechanisms.

Polyphenol/Source	Condition/Model	Impact on Microbiota and Associated Mechanisms	Ref.
Preclinical data			
Epicatechin gallate	In vitro assay in bacterial medium	Sensitizes methicillin-resistant *S. aureus* to beta-lactam antibiotics	[74]
Green tea and red wine polyphenols	In vitro assay in bacterial medium	Inhibits the VacA toxin, a key virulence factor of *Helicobacter pylori*	[75]
Quercetin	High fat diet (animal model)	Reduction of BW. Decrease *Firmicutes* populations, Erysipelotrichi class and *Bacillus* genus. Down-regulation of *Erysipelotrichaceae*, *Bacillus* and *Eubacterium cylindroides*	[76]
Proanthocyanidin rich red wine extract	Colon cancer (animal model)	Treated rats exhibited considerably lower levels of *Clostridium* spp. and higher levels of *Bacteroides*, *Lactobacillus* and *Bifidobacterium* spp.	[77]
Coffee and Caffeic acid	Colon cancer (animal model)	Intake precisely inhibited colon cancer metastasis and neoplastic cell transformation in mice by inhibiting TOPK (T-LAK cell-originated protein kinase) and MEK1	[78]
Resveratrol	Colonic cancer (animal model)	Reduced activities of faecal and host colonic mucosal enzymes, such as α-glucoronidase, nitroreductase, β-galactosidase, mucinase, and α-glucosidase	[79]
Resveratrol	DSS induced colitis (animal model)	Stimulated faecal cell counts of *Lactobacillus* and *Bifidobacterium* spp.	[80]
Polyphenols (from plants)	In vitro assay in bacterial medium	Control of food-borne pathogenic bacteria without inhibitory effect on lactic acid bacteria growth	[81]
Polyphenols (from algae)	In vivo assay in TD2M mice	Hypoglycemic effect together with decreased counts of Turcibacter and *Akkermansia* and increase of *Alistipes*	[82]
Polyphenols (Chinese propolis, Brasilian propolis)	DSS induced colitis (animal model)	Modulation of the GM composition, namely reduction of the *Bacteroides* spp.	[83]
Polyphenols (*Prunella vulgaris* honey)	DSS induced colitis (animal model)	Modulation of GM composition, with increased Bacteroidetes/Firmicutes ratio and restoration of *Lactobacillus* spp. populations	[84]
Polyphenols (from fungi)	DSS induced colitis (animal model)	Modulation of GM composition, with reduction of Firmicutes/Bacteroidetes ratio and restoration of *Lactobacillus* spp. populations	[85]
Human studies			
(+)Catechin and (−)Epicatechin	In vitro assay with faecal samples of healthy volunteers	Inhibition of *Clostridium histolyticum* growth and boosted the growth of members of the *Clostridium coccoides*-*Eubacterium rectale* group and *E. coli*, while growth of *Lactobacillus* Spp. and *Bifidobacterium* Spp. remained comparatively unaltered	[86]
Proanthocyanidin rich grape extract	Fecal flora and odor (healthy adults	Significantly increase in the number of *Bifidobacteria*	[87]
Cocoa-derived flavanols	Healthy humans	Stimulate growth and proliferation of *Bifidobacterium* spp. and *Lactobacillus* spp., together with reduction in plasma C-reactive protein (CRP)	[71]
Polyphenols (Red wine)	Human study	Regular intake results in BP reduction, lipid profile improvement (e.g., TGs) and decline in uric acid levels, together with increase in the proliferation of *Bacteroides* spp.	[88]
Polyphenols (Green tea, fruits, vinegar wine)	Obese volunteers	Weight lowering effect together with alteration in gut microflora	[89]
Dihydroxylated phenolic acid	In vitro LPS-induced inflammation	Exhibits potent anti-inflammatory properties, lowering the secretion of TNF-α, IL-1b and IL-6 in LPS-induced peripheral blood mononuclear cells from healthy individuals	[90]
Polyphenols (from spices)	Healthy humans	Glucose uptake and appetite modulation	[91]

## Abbreviations

AGEs	advanced glycation end products
AP-1	activator protein 1
APCs	antigen presenting cells
CAP	cellulose acetate phthalate
CD	Crohn disease
COX-2	cyclooxygenase-2
CRC	colorectal cancer
DN	diabetic nephropathy
DR	diabetic retinopathy
DSS	dextran sulfate sodium
GI	gastrointestinal
GM	gut microbiota
GRAS	generally recognized as safe
HPMCP	hydroxyl propyl methylcellulose phthalate
HSPs	heat shock proteins
IBD	inflammatory bowel diseases
IL6	interleukin 6
IS	indoxyl sulfate
LPS	lipopolysaccharide
MAMP	microbe-associated molecular pattern
VEGF	vascular endothelial growth factor
NF-kB	nuclear factor kappa-light-chain-enhancer of activated B cells
PC	p-cresol
PCS	p-cresyl sulfate
PG	peptidoglycan
PYY	peptide tyrosine-tyrosine
SCFAs	short-chain fatty acids
TMAO	trimethylamine-N-oxide
TNF-α	tumor necrosis factor-alpha
TOPK	T-LAK cell-originated protein kinase
TLRs	toll-like receptors
UC	ulcerative colitis
